# ﻿*Leptanillavoldemort* sp. nov., a gracile new species of the hypogaeic ant genus *Leptanilla* (Hymenoptera, Formicidae) from the Pilbara, with a key to Australian *Leptanilla*

**DOI:** 10.3897/zookeys.1197.114072

**Published:** 2024-04-11

**Authors:** Mark K. L. Wong, Jane M. McRae

**Affiliations:** 1 School of Biological Sciences, The University of Western Australia, Crawley, WA 6009, Australia The University of Western Australia Crawley Australia; 2 Centre for Environment and Life Sciences, Commonwealth Scientific and Industrial Research Organization, Floreat, WA 6014, Australia Centre for Environment and Life Sciences, Commonwealth Scientific and Industrial Research Organization Floreat Australia; 3 Bennelongia Environmental Consultants, 5 Bishop Street, Jolimont, WA 6014, Australia Bennelongia Environmental Consultants Jolimont Australia

**Keywords:** Australia, hypogaeic, Leptanilla, Milieu Souterrain Superficiel, subterranean

## Abstract

The genus *Leptanilla* Emery, 1870 of the family Formicidae, subfamily Leptanillinae, comprises miniscule, pale, blind ants that are rarely collected and poorly understood due to their hypogaeic (i.e. underground) lifestyles. Here we describe a new *Leptanilla* species from two workers collected via subterranean scraping in the arid Pilbara region of Western Australia. *Leptanillavoldemort***sp. nov.** is the second leptanilline species documented in Australia after the elusive *Leptanillaswani* Wheeler, 1932. Workers of *L.voldemort***sp. nov.** display a remarkably gracile morphology characterised by elongated legs, antennae, and mandibles, and they are easily differentiated from other *Leptanilla* species. We also provide new measurements *for L.swani* from two workers found proximally to the type locality of *L.voldemort***sp. nov.** A key to the worker caste of *Leptanilla* species of the Australian continent is presented.

## ﻿Introduction

Although lacking the impressive colours, armoury and colony sizes seen in many of the world’s ~14,000 ant species (Bolton 2024), the diminutive members of the genus *Leptanilla* Emery, 1870 (Formicidae, Leptanillinae) are darlings of myrmecologists, for they include some of the most elusive and bizarre ants on Earth. All known *Leptanilla* species are hypogaeic, living in small colonies that nest and forage exclusively underground (Griebenow 2024; Qian et al. 2024). As such, individuals of *Leptanilla* are rarely collected by conventional methods for sampling ants (e.g. pitfall traps), which tend to target surface habitats (Wong and Guénard 2017). In adaption to subterranean life, *Leptanilla* ants characteristically display cryptobiotic morphological traits; their workers are very small (often <2 mm), depigmented and blind (Bolton 1990; Wong and Guénard 2016). The few observations of live *Leptanilla* colonies have also revealed intriguing, specialised behaviours, such as specialised predation on geophilomorph centipedes (Masuko 1990), and adult feeding on haemolymph (i.e. blood) of the larvae from a unique duct-like organ in the larval integument—the “larval haemolymph tap” (Masuko 1989). Moreover, recent phylogenomic analyses show that together with the monotypic Martialinae, the Leptanillinae constitute the sister group to all other extant ant species, making them especially significant for our understanding of ant evolution (Borowiec et al. 2019; Romiguier et al. 2022; but see Cai 2024).

The 61 described species of *Leptanilla* are found in tropical and temperate regions of the Old World (Bolton 1990; Griebenow 2024; Qian et al. 2024). The apparently patchy distribution of the genus may be an artefact of geographic biases in ant research in general (Kass et al. 2022) and the use of subterranean sampling techniques in particular (Wong and Guénard 2017). While multiple *Leptanilla* species have been documented in Africa, Europe, and Asia, only one species, *Leptanillaswani* Wheeler, 1932 has been described from Australia thus far. Moreover, few if any workers of *L.swani* have been found in the 90 years since it was first described (Wheeler 1932; Heterick 2022; AntWeb 2024). Although winged *Leptanilla* males have been collected in flight-interception traps throughout the continent, the identities of these are questionable, as none occur in sympatry with definitive workers of *L.swani* (AntWeb 2024; Zachary Griebenow pers. comm.).

Here, we describe a new *Leptanilla* species, *Leptanillavoldemort* sp. nov., from two workers collected in the arid Pilbara region of Western Australia. *Leptanillavoldemort* sp. nov. represents the second species of *Leptanilla* and second member of the subfamily Leptanillinae from the Australian continent. Notably, workers of *L.voldemort* sp. nov. display a distinctly gracile morphology, characterised by elongated legs, antennae, and mandibles. This remarkable phenotype is not seen in most other *Leptanilla* species – including the other Australian species, *L.swani*, for which new morphological measurements are hereby reported from two workers collected proximally to the type locality of *L.voldemort* sp. nov. A key to the worker caste of *Leptanilla* from Australia is also presented.

## ﻿Methods

Photographs of specimens were obtained with an incorporated digital camera mounted on a Leica M205C dissecting microscope through the Leica Application Suite V4 software. A total of 33–72 images were taken and stacked together. Morphological measurements and indices were calculated following Griebenow et al. (2022). These are detailed below.

**HW** (head width): maximum width of cranium in full-face view.

**HL** (head length): maximum length of head in full-face view from anterior margin of head to cranial vertex.

**SL** (scape length): maximum length of scape in medial view, excluding bulbus.

**MaL** (mandible length): maximum length of mandible from view orthogonal to lateral mandibular margin, measured from ventral mandibular articulation to mandibular apex.

**WL** (Weber’s length): maximum diagonal length of mesosoma in profile view, measured from most anterior extent of pronotum excluding cervical shield to most posterior extent of propodeal lobes, when present.

**PrW** (pronotal width): maximum width of pronotum, measured in dorsal view.

**MW** (mesonotal width): maximum width of mesonotum in dorsal view, measured immediately anterior to mesocoxal foramen.

**PTL** (petiolar length): maximum length of petiole in dorsal view, not including presclerites.

**PTH** (petiolar height): maximum height of petiole in profile view, including sternal process and dorsal node, if distinct.

**PTW** (petiolar width): maximum width of petiole in dorsal view.

**PPL** (postpetiolar length): maximum length of postpetiole in dorsal view, not including presclerites.

**PPW** (postpetiolar width): maximum width of postpetiole in dorsal view.

**PPH** (postpetiolar height): maximum height of postpetiole in profile view, including sternal process and dorsal node, if distinct.

**CI** (cephalic index) = HW ÷ HL × 100.

**SI** (scape index) = SL ÷ HW × 100.

**MI** (mandibular index) = MaL ÷ HW × 100.

**PI** (petiolar index) = PTW ÷ PTL × 100.

**PPI** (postpetiolar index) = PPW ÷ PPL × 100.

**PPHI** (postpetiolar height index) = PPH ÷ PPL × 100.

Abbreviations of the type depositories are as follows:

**ANIC**Australian National Insect Collection;

**WAM**Western Australian Museum.

## ﻿Results

### ﻿Species accounts

#### ﻿ Leptanilla voldemort

Taxon classificationAnimaliaHymenopteraFormicidae

Wong & McRae sp. nov.

833CD508-3201-5660-9EE0-F26C34C5FE4C

https://zoobank.org/6C23DA06-A060-4326-A318-991C7EB3C7A1

Figs 1 , 2 , 3 , 4

##### Type material.

***Holotype*.** Australia • 1 worker; Western Australia, Newman; 22°44'S, 119°02'E; ca 575 m a.s.l.; 8 Mar. 2023; Jane M. McRae leg.; collected via subterranean scraping; BENNSPECIMENID_746962.1; WAM.

***Paratype*.** Australia • 1 worker; same data as for holotype; BENNSPECIMENID _746962; WAM.

**Figure 1.**
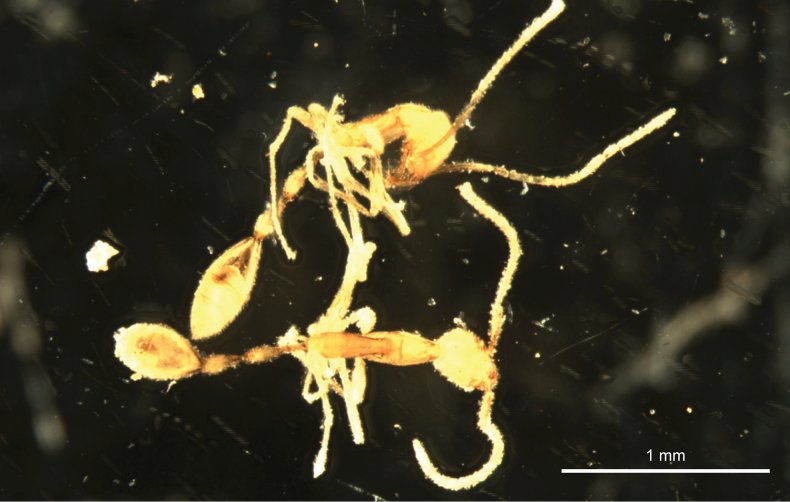
Workers of *Leptanillavoldemort* sp. nov. from Western Australia in ethanol.

**Figure 2.**
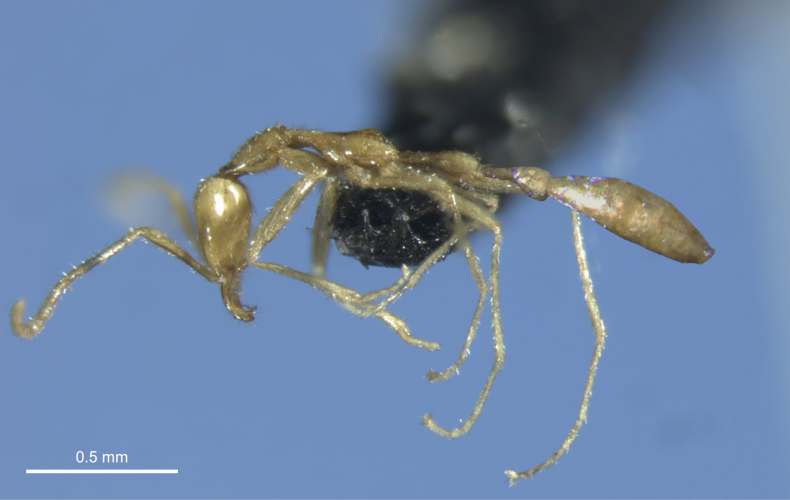
Profile view of *Leptanillavoldemort* sp. nov. (holotype) from Western Australia. The postpetiole and gaster of the specimen, which were disconnected from the main body during mounting, were imaged separately and subsequently attached to the body digitally while ensuring consistency of scale.

**Figure 3.**
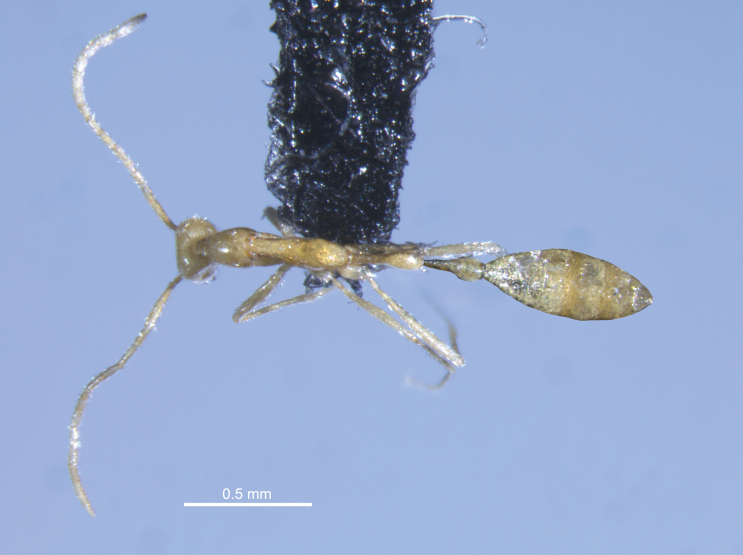
Dorsal view of *Leptanillavoldemort* sp. nov. (holotype) from Western Australia. The postpetiole and gaster of the specimen, which were disconnected from the main body during mounting, were imaged separately and subsequently attached to the body digitally while ensuring consistency of scale.

Unfortunately, both the holotype and paratype specimens were brittle and partially damaged during the mounting process. A photograph of the fully intact specimens in liquid prior to mounting is shown in Fig. 1. During mounting of the holotype, the postpetiole was disconnected from the petiole and gaster. The paratype was similarly disconnected at the petiole and gaster. Broken segments of each specimen were glued onto its respective mount. The full-body images of the mounted holotype in profile view (Fig. 2) and dorsal view (Fig. 3) are composites in which the postpetiole and gaster were imaged separately in the respective views, and subsequently reattached to the body digitally, while ensuring consistency of scale.

##### Measurements and indices.

All measurements are in millimetres (mm).

***Holotype***: HW 0.26; HL 0.35; SL 0.36; MaL 0.21; WL 0.59; PrW 0.16; MW 0.12; PTL 0.28; PTH 0.08; PTW 0.07; PPL 0.24; PPW 0.10; PPH 0.10; CI 73, SI 139, MI 81, PI 25, PPI 39, PPHI 42.

***Paratype* (*n* = 1)**: HW 0.27; HL 0.36; SL 0.35; MaL 0.20; WL 0.61; PrW 0.16; MW 0.12; CI 75, SI 128, MI 75.

##### Worker description.

**Figure 4.**
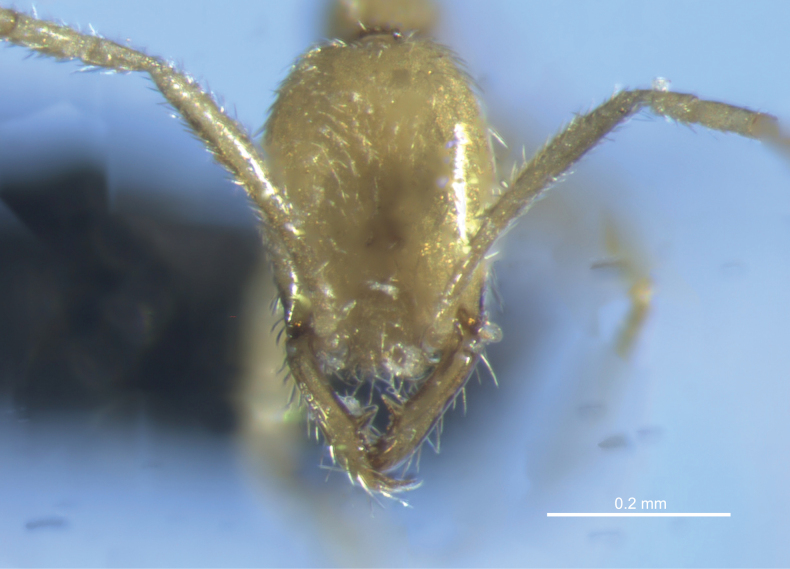
Full-face view of *Leptanillavoldemort* sp. nov. (holotype) from Western Australia.

***Head*.** Head longer than wide (CI = 73–75). In full-face view (Fig. 4), posterior margin of head slightly concave. Lateral margins of head slightly convex. Eyes absent. Anterior clypeal margin extending forward with two rounded lobes anterolaterally and slightly concave on its anteromedian portion. Median portion of clypeus raised; frontoclypeal process present and concealing labrum. Mandibles long relative to head (MI = 75–81) and armed with three teeth. Apical tooth acute and larger than subapical and basal teeth. Basal tooth larger than subapical tooth with tip approximately perpendicular to mandibular margin; margin distal to subapical tooth irregularly serrate. Antennal insertion exposed. Antennae with 12 segments. Scape elongated, extending well beyond mid-point of head (SI = 128–139); margins subparallel, expanding slightly before tapering at apex. Pedicel longer than broad and constricted at separation from scape; constriction separating pedicel from flagellum not pronounced. Flagellum filiform; all flagellomeres longer than broad. Antennomere 12 approximately double the length of previous flagellomeres with apex tapered.

***Mesosoma*.** In dorsal view, maximum width of pronotum (PrW = 0.16 mm) wider than posterior portions of mesosoma (Fig. 3). In lateral view, pronotal dorsum slightly convex, tapering along anterior margin, with posterior margin slightly elevated above mesonotal dorsum (Fig. 2). Promesonotal suture clearly visible in both lateral and dorsal view. In dorsal view, mesonotum constricted anteriorly, with lateral margins gently convex and approximating maximum width at fusion with propodeum. In dorsal view, propodeum not constricted anteriorly, with outline of posterior margin trapezoidal (Fig. 3). In lateral view, propodeal dorsum raised and strongly convex, with posterior forming a strongly convex propodeal declivity at an approximate 65° angle (Fig. 2). Metapleural gland bulla and propodeal spiracle visible. Coxae robust, pro- and mesocoxae well separated; distal leg articles elongated.

***Metasoma*.** Metasoma elongated in both dorsal and lateral view (PL + PPL ≈ WL). In dorsal view (Fig. 3), petiole four times as long as wide (PI = 25), with lateral margins subparallel at anterior and convex after mid-point to achieve maximum width; posterior margin convex and rounded. Postpetiole longer than wide (PPI = 39), with similar shape to petiole but wider and more rounded posteriorly. In lateral view (Fig. 2), petiole with dorsal and ventral margins subparallel at anterior and convex after mid-point to achieve maximum height; posterior margin slightly concave. Subpetiolar process absent. Postpetiole with dorsal and ventral margins subparallel at anterior and diverging near mid-point, after which dorsal margin is weakly convex and ventral margin is strongly concave; posterior margin concave.

***Sculpture*.** Sculpture absent. Most of the body slick and shiny (i.e. not a result of glare from diffusing light when imaging).

***Pubescence*.** Pubescence present on most of the body, especially antennae and legs, but sparse to absent on propodeum and metasoma. Numerous suberect to erect setae on dorsal and ventral surfaces of pronotum, cranium, and mandibles. Long basal and subapical setae on mandibles.

***Colouration*.** Pale gold to amber. Colouration slightly lighter at extremities.

***Castes*.** Male and gyne unknown.

##### Etymology.

The species epithet pays tribute to the antagonist in the Harry Potter book series, Lord Voldemort, a terrifying wizard who, like the new ant, is slender, pale, and thrives in darkness. The species epithet is a noun, and thus invariant.

##### Distribution.

Only known from the type locality within the Pilbara region of Western Australia.

##### Ecology.

*Leptanillavoldemort* sp. nov. was collected from a hot grassland in the north-west Pilbara, a region characterised by very hot summers (average maximum 36–39 °C), low winter minima (average minimum 6–12 °C), low average annual rainfall (200–350 mm), and high evaporation (average annual potential evaporation 3200–4000 mm) (Eberhard et al. 2005). Both type specimens were collected from a 25 m deep mining exploration drill hole using a subterranean scraping method, whereby a weighted net was lowered to the base of the hole and dragged four times back to the surface against the wall of the hole (Halse and Pearson 2014). The drill hole was in a dry drainage line, with the subterranean substrate consisting of coarse alluvium near surface over banded iron formation at depth. Other organisms recorded from the drill hole include troglofaunal beetles of an unknown genus, troglofaunal flies of the genus *Allopnyxia* Freeman, 1952 and troglofaunal centipedes assigned to the genus *Cryptops* Leach, 1814. We are presently unable to ascertain whether colonies of *L.voldemort* sp. nov. inhabit topsoil, the subsurface alluvium, or voids in the deeper weathered banded iron formation. The colony size and structure of *L.voldemort* sp. nov. is unknown.

##### Remarks.

The worker of *L.voldemort* sp. nov. is easily distinguished from the other native Australian leptanilline species, *L.swani* Wheeler, 1932, which is evidently sympatric with *L.voldemort* sp. nov. (see new collection data below). First, *L.voldemort* sp. nov. has distinctly elongated mandibles (MI = 75–81) and antennae (SI = 128–139), while in *L.swani* these appendages are stouter and shorter (MI = 44–56, SI = 59–74). Second, *L.voldemort* sp. nov. possesses metasomal segments that are two to four times longer than wide (PI = 25, PPI = 39), while in *L.swani* these segments are almost as long as wide (PI = 56–70, PPI = 83–100). Finally, *L.voldemort* sp. nov. (WL = 0.59–0.61 mm) is larger in size than *L.swani* (WL = 0.35–0.45 mm). In general, the gracile phenotype of *L.voldemort* sp. nov. is distinctive among the genus *Leptanilla*, except for *Leptanillalaventa* Griebenow, Moradmand & Isaia, 2022, a species described from Iran. Specifically, the elongated antennae and petiole of workers in both *L.voldemort* sp. nov. (SI = 128–139, PI = 25) and *L.laventa* (SI = 160–163, PI = 29–32) are not observed in other *Leptanilla* species (SI<100, PI>31) (Griebenow et al. 2022; Griebenow 2024; Qian et al. 2024). Nonetheless, workers of *L.voldemort* sp. nov. can be distinguished from those of *L.laventa* based on several key morphological differences. First, in dorsal view, the shape of the petiole and postpetiole of *L.voldemort* sp. nov. is distinctly more elongated (PI = 25; PPI = 39) than in *L.laventa* (PI = 29–32; PPI = 59–64.7). Second, in lateral view, the propodeal declivity of *L.voldemort* sp. nov. is strongly convex and distinctly angular, whereas that of *L.laventa* is weakly convex and gently rounded. Third, in full-face view, the axis of the basal mandibular tooth of *L.voldemort* sp. nov. extends almost perpendicular to the mandibular margin, with the tip of the tooth forming an 80–90° angle with the medial mandibular margin, whereas in *L.laventa*, the basal tooth is recurved, with the tip of the tooth forming a 60–70° angle with the medial mandibular margin. Finally, *L.voldemort* sp. nov. (WL = 0.59–0.61 mm) is smaller in size than *L.laventa* (WL = 0.74–0.85 mm).

#### ﻿ Leptanilla swani

Taxon classificationAnimaliaHymenopteraFormicidae

Wheeler, 1932

D4887DC9-A1FF-52A8-9788-938B14D7515F

##### Notes.

Below we provide measurements for three worker specimens of *L.swani*. Collection data for the first two specimens are as follows: Australia; 2 workers; Western Australia, Newman; 22°47'S, 119°9'E; ca 537 m a.s.l.; 9 May 2022; Jane M. McRae leg.; collected via subterranean scraping; BENNSPECIMENID_735794 and BENNSPECIMENID_735840; WAM. Collection data for the third specimen, a paratype of the species, is as follows: ***Paratype***: Australia; 1 worker; Western Australia, Chittering; 31°27'S, 116°5'E; ca. 225 m a.s.l.; D. Swan leg.; CASENT0172006; ANIC.

**Figure 5.**
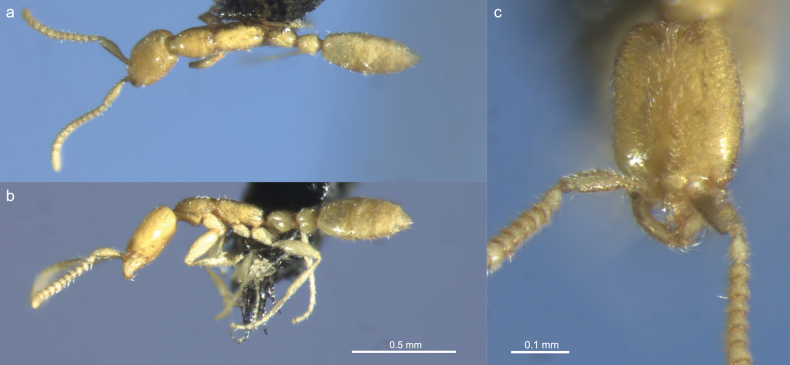
Worker of *Leptanillaswani* (BENNSPECIMENID_735794) **a** dorsal view **b** profile view **c** full-face view.

BENNSPECIMENID_735794 (Fig. 5): HW 0.23; HL 0.31; SL 0.17; MaL 0.13; WL 0.44; PrW 0.15; MW 0.14; PTL 0.15; PTH 0.11; PTW 0.08; PPL 0.11; PPW 0.09; PPH 0.13; CI 72, SI 74, MI 56, PI 56, PPI 83, PPHI 117.

BENNSPECIMENID_735840: HW 0.22; HL 0.31; SL 0.16; MaL 0.12; WL 0.45; PrW 0.15; MW 0.12; PTL 0.15; PTH 0.11; PTW 0.09; PPL 0.12; PPW 0.10; PPH 0.12; CI 71, SI 72, MI 55, PI 59, PPI 87, PPHI 106.

CASENT0172006: HW 0.20; HL 0.28; SL 0.12; MaL 0.09; WL 0.35; PrW 0.13; MW 0.11; PTL 0.11; PTH 0.10; PTW 0.08; PPL 0.08; PPW 0.08; PPH 0.10; CI 69, SI 59, MI 44, PI 70, PPI 100, PPHI 125.

### ﻿Synoptic species list of Australian *Leptanilla* species

*Leptanillaswani* Wheeler, 1932

*Leptanillavoldemort* sp. nov.

### ﻿Key to Australian *Leptanilla* species based on the worker caste

**Table:** 

1	Elongated mandibles (MI = 75–81), antennae (SI = 128–139), and metasoma (PI = 25, PPI = 39). Large body size (WL = 0.59–0.61)	***L.voldemort* sp. nov.**
−	Stout mandibles (MI = 44–56), antennae (SI = 59–74), and metasoma (PI = 56–70, PPI = 83–100). Small body size (WL = 0.35–0.45)	***L.swani* Wheeler**

## ﻿Discussion

Despite Australia being a global hotspot for ant diversity (Kass et al. 2022), *Leptanillavoldemort* sp. nov. is only the second species of *Leptanilla* and second member of the subfamily Leptanillinae known from the continent since the discovery of *L.swani* in 1932. To this end, it is envisaged that the targeted employment of currently underutilised techniques for collecting hypogaeic ant species such as subterranean scraping, subterranean pitfall traps, and soil extractions (Wong and Guénard 2017) may facilitate the discovery of additional Australian leptanilline species. The Pilbara region of north-western Australia, in particular, is one of the oldest land surfaces on Earth (Pepper et al. 2008). Due to multiple vicariant isolation and divergence events that have occurred with surface aridification, this region harbours globally significant levels of endemic diversity in subterranean invertebrate species (Mokany et al. 2019). The presence of both *L.swani* and *L.voldemort* sp. nov. in the Pilbara highlights the importance of this region for Australian leptanilline species.

Although we collected individuals of both *L.voldemort* sp. nov. and *L.swani* within the same general locality (sites <15 km apart) in the Pilbara, the two sympatric species clearly exhibit contrasting morphologies. Whereas *L.swani* is stout and compact (Fig. 5), *L.voldemort* sp. nov. is distinctly gracile (Fig. 2). We posit that these morphological differences may relate to the species’ use of dissimilar microhabitats within the subterranean environment. Given that a colony of *L.swani* was collected from under a large stone (Wheeler 1932), it is reasonable to assume that *L.swani* inhabits the shallower layers of soil. Moreover, the generally compact body plan of *L.swani* resembles that of most other *Leptanilla* species that have been collected via the sifting of topsoil (10–20 cm depth) or via the installation of subterranean pitfall traps (10–50 cm depth) in the soil layer (Wong and Guénard 2016, 2017). In contrast, the distinctively gracile morphology of *L.voldemort* sp. nov. is unlike that of any other *Leptanilla* species except *L.laventa* from Iran. Interestingly, *L.laventa* was not collected from soil, but via subterranean pitfall traps buried 0.6–1 m belowground in the *Milieu Souterrain Superficiel*, an underground network of empty air-filled voids and cracks developing within multiple layers of rock fragments, located below the soil layer (Mammola et al. 2016; Griebenow et al. 2022). We can only presently speculate whether *L.voldemort* sp. nov. occupies a similar microhabitat; systematic sampling of the ants from different vertical sections of the subterranean column (via subterranean pitfall traps or excavations) coupled with measurements of climatic and structural parameters of the substrate should provide clarification on the matter. Finally, the elongated mandibles and large, sharp basal teeth of *L.voldemort* sp. nov. suggest that the ants are likely specialised predators; however, it remains to be seen whether they prey on geophilomorph centipedes, as in other species of *Leptanilla* (Masuko, 1990), or other hypogaeic taxa, such as centipedes of the family Cryptopidae (Chilopoda, Scolopendromorpha), hypogaeic cockroaches, and pauropods, all of which were collected from the same drill hole as *L.voldemort* sp. nov.

## Supplementary Material

XML Treatment for Leptanilla voldemort

XML Treatment for Leptanilla swani

## ﻿Additional information

### Author ORCIDs

Mark K. L. Wong https://orcid.org/0000-0002-6248-3103

### Data availability

All of the data that support the findings of this study are available in the main text.
